# Novel Hybrid Approach to Portal Vein Thrombectomy Immediately Prior to Orthotopic Liver Transplantation

**DOI:** 10.1155/crit/8882747

**Published:** 2025-11-29

**Authors:** Jonathan Jou, Brandon Pearson, Zachary Haber, Samer Ebaid

**Affiliations:** Department of Liver Transplantation and Hepatobiliary Surgery, University of California Los Angeles, Los Angeles, California, USA

## Abstract

Portal vein thrombosis (PVT) is common among liver transplant candidates, affecting up to 26% of patients awaiting liver transplant. Despite the suspected negative impact of PVT on liver transplant outcomes, consensus recommendations remain lacking. Thrombus circumvention is most commonly achieved with eversion portal thromboendovenectomy or venous jump graft. We present a novel hybrid approach addressing PVT in a patient with acute hepatic decompensation and worsening clot burden extending to the superior mesenteric and splenic veins. This patient underwent a transjugular intrahepatic portosystemic shunt (TIPS) placement and endovascular thrombectomy of the splanchnic veins less than 24 h prior to undergoing an orthotopic liver transplant with the graft maintained on normothermic machine perfusion. Three-month postoperative scans demonstrated a patent portal venous system with trace residual clot burden. This is the first reported case utilizing a hybrid approach for a portal vein thrombus extending into the superior mesenteric and splenic veins with subsequent orthotopic liver transplant.

## 1. Introduction

Portal vein thrombosis (PVT) is a common complication among patients with end-stage liver disease, occurring in up to 26% of those awaiting liver transplantation. Although PVT is suspected to adversely affect transplant outcomes, there is a lack of consensus regarding optimal management strategies, particularly in complex cases. Traditional surgical approaches for thrombus management during liver transplantation include eversion portal thromboendovenectomy and the use of venous jump grafts to bypass the thrombosed segment. However, these techniques may be inadequate or technically challenging in the presence of an extensive thrombus burden.

We describe a novel hybrid strategy combining preoperative endovascular and surgical interventions to address extensive PVT in a patient with acute hepatic decompensation and progressive thrombosis involving the portal vein, superior mesenteric vein (SMV), and splenic vein. This approach included transjugular intrahepatic portosystemic shunt (TIPS) placement and endovascular thrombectomy within 24 h prior to orthotopic liver transplantation (OLT), with the graft preserved using normothermic machine perfusion. To our knowledge, this is the first reported case employing such a multidisciplinary approach for extensive splanchnic venous thrombosis in the setting of urgent OLT.

## 2. Case Report

**Figure 1:**
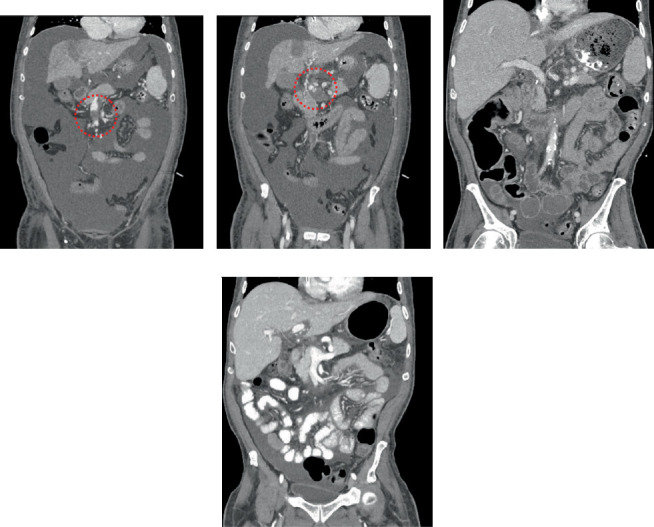
Computed tomography scans pre- and postintervention. (a, b) Scan demonstrating portal vein thrombus extending into the SMV and splenic vein confluence (red dotted circles). (c, d) Three months posttransplant demonstrating trace clot burden and patent splanchnic venous vasculature.

A 72-year-old male with chronic liver failure secondary to metabolic dysfunction associated with steatohepatitis complicated by esophageal and gastric variceal bleeding, ascites, and a 2.2-cm Segment 4A/B hepatocellular carcinoma status post Yttrium-90 ablation 2 weeks prior presented to the intensive care unit for acute decompensation. Model of end-stage liver disease score was calculated at 40. Computed tomography (CT) scan performed on admission demonstrated no new lesions and a nonocclusive bland thrombus of the main portal vein, SMV, and splenic vein (Figure 1). Thrombus extent was concerning for flow limitation of the portal vein that may prevent successful perfusion of a hepatic graft. The decision was made to consult interventional radiology (IR) and plan for TIPS placement and endovascular portal vein thrombectomy to be performed at the time of organ offer acceptance.

**Figure 2:**
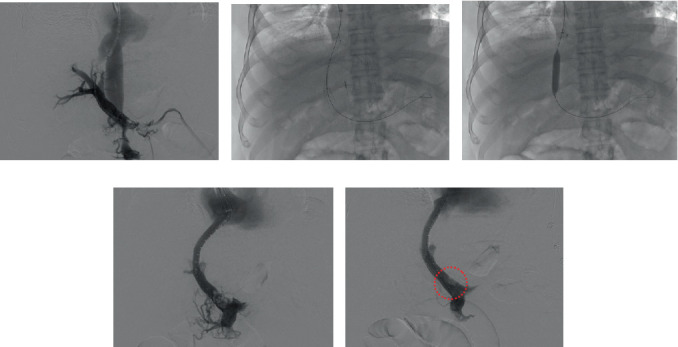
TIPS and endovenous thrombectomy. (a) Venogram demonstrating patent IVC and thrombus burden within portal, SMV, and splenic veins. (b, c) 10 × 90 mm TIPS deployment and balloon dilation. (d) Advancement of Inari Triever16 and Triever20 mechanical and aspiration thrombectomy device. (e) Completion venogram demonstrating small residual filling defect within proximal main portal vein (red dotted circle).

An organ was accepted from a 43-year-old donor declared brain dead 4 days prior with an in-house backup recipient arranged in case the thrombectomy proved unsuccessful. Due to the need for an intravascular procedure without guaranteed success prior to transplant, the decision was made to place the organ on a normothermic machine perfusion pump to remove the time pressure associated with static cold storage. The patient underwent a successful TIPS with access into the portal vein as well as a thromboaspiration of the portal vein, SMV, and splenic vein with improved flow and filling deficits without evidence of significant flow limitation on postaspiration venography (Figure 2). He underwent an OLT the following morning.

Donor cross-clamp time was 1822 and the organ was placed on normothermic machine perfusion. The hepatectomy was performed in standard fashion with preservation of the native inferior vena cava (IVC). The portal vein was opened during the anhepatic phase to allow for removal of additional clots from the SMV and splenic vein using a Fogarty catheter. The hepatic graft was implanted in standard piggyback fashion with a suprahepatic anastomosis to the hepatic veins and a stapled donor infrahepatic IVC closure. Reperfusion time was 0947. Arterial reconstruction was completed in the standard fashion. Medistim measurements demonstrated 1600 mL/min portal and 300 mL/min arterial flow. The choledochocholedochostomy with internal stent was created for size discrepancy. At the conclusion of the case, 9 units of packed red blood cells, 11 units of fresh frozen plasma, 1 unit of cryoprecipitate, and 1 unit of platelets were transfused.

Postoperative bleeding prompted a repeat CT scan 4 days after his index operation demonstrating a 10-cm right posterior perihepatic hematoma with several foci of active extravasation and improved nonocclusive thrombi of the SMV and proximal portal/splenic vein confluence. He returned to the operating room where bleeding from one of the short hepatic veins close to the infrahepatic IVC was oversewn. Repeat Medistim measurements of the portal vein demonstrated more than 2000 mL/min of portal flow. He was discharged to an acute rehabilitation facility 32 days after his index operation and discharged home to his family 11 weeks after his acute rehabilitation stay. Renal recovery and appropriate weight gain with a gastrostomy tube placed 6 weeks postoperatively were achieved 3 months after his index operation, and his hemodialysis catheter and gastrostomy tube were removed.

CT scan 3 months after transplant demonstrated trace residual nonocclusive thrombus within the central SMV (Figure 1c,d). Explant pathology demonstrated moderately differentiated hepatocellular carcinoma of Segment 4A/4B with less than 1 cm of viable tumor and 95% tumor necrosis. Oncology recommended routine imaging surveillance and no adjuvant chemotherapy.

## 3. Discussion

PVT is a significant challenge in liver transplantation, occurring in up to 26% of cirrhotic patients [1]. Thromboendovenectomy is considered the procedure of choice for PVT management; however, the use of jump grafts in cases of thrombus extension beyond the portal vein has also been reported [2–5]. The use of endovascular approaches for extensive PVT has been limited in the literature, with recent publications suggesting TIPS and direct or indirect thrombolysis result in significant clinical improvement with a risk of rethrombosis between 1 and 4 months and bowel ischemia requiring resection [6, 7].

While sequelae of cirrhosis usually manifest as bleeding secondary to portal hypertension, recent studies suggest that cirrhosis may also portend hypercoagulability despite elevated international normalized ratio [8, 9]. The degree of cirrhosis is thought to physically contribute to higher rates of portal vein thrombus due to decreased portal flow velocity [10]. This risk may increase with inflammatory disorders such as pancreatitis or intra-abdominal infections that disrupt the endothelium [11]. Obesity, metabolic syndrome, and visceral fat have also been associated with the presence of PVT [12]. PVT may be detected on surveillance ultrasounds, but cross-sectional imaging with CT scan is recommended to confirm the diagnosis and evaluate clot burden [13], which may facilitate improved preoperative planning.

Patients with prolonged compensated cirrhosis, longstanding portal hypertension, and liver cancer may be at higher risk for thrombus formation. While the presence of PVT was a contraindication to transplant in the past, advancements in technique have allowed for clinical judgment to supersede dogma. The only true contraindication is suppurative PVT, usually secondary to an acute intra-abdominal infection [13].

Transplant outcomes for < 50% PVT are comparable to non-PVT patients, whereas outcomes for completely obstructed portal vein thromboses carry as much as a 50% decrease in survival at 1 year [14]. Despite the suspected negative impact of PVT on liver transplant outcomes, rigorous studies and consensus recommendations remain lacking. This is the first reported case utilizing a hybrid approach between IR and transplant surgery to address a portal vein thrombus extending into the SMV and splenic vein with immediate subsequent OLT.

The completion of a TIPS and IR thrombectomy just prior to transplantation was made more feasible with the decision to place the organ on normothermic machine perfusion. Due to the chronic nature of the thrombus without worsening clinical signs, the decision was made not to pursue thrombectomy until an organ offer was actually accepted. Warm perfusion of the graft allowed our in-house team time to complete the thrombectomy without the time pressure associated with static cold storage. It also created an opportunity for the liver to be provided to a backup donor in case the IR procedure was unsuccessful. This case demonstrates that a combined strategy employing an immediate TIPS, thrombectomy, and normothermic perfusion graft may be an adjunct to traditional open thrombectomy especially in the event of extensive PVT in order to restore appropriate portal flow to the graft.

## Data Availability

The data that support the findings of this study are available on request from the corresponding author. The data are not publicly available due to privacy or ethical restrictions.
